# Injury-Induced HSP27 Expression in Peripheral Nervous Tissue Is Not Associated with Any Alteration in Axonal Outgrowth after Immediate or Delayed Nerve Repair

**DOI:** 10.3390/ijms22168624

**Published:** 2021-08-11

**Authors:** Lena Stenberg, Derya Burcu Hazer Rosberg, Sho Kohyama, Seigo Suganuma, Lars B. Dahlin

**Affiliations:** 1 Department of Translational Medicine—Hand Surgery, Lund University, 205 02 Malmö, Sweden; derya_burcu.hazer_rosberg@med.lu.se (D.B.H.R.); lars.dahlin@med.lu.se (L.B.D.); 2 Department of Neurosurgery, Faculty of Medicine, Mugla Sıtkı Kocman University, Mugla 48100, Turkey; 3 Department of Orthopaedic Surgery, Faculty of Medicine, University of Tsukuba, 1-1-1 Tennodai, Tsukuba 305-8575, Japan; sho_kohyama_1025@yahoo.co.jp; 4 Department of Orthopaedic Surgery, Ishikawa Prefectural Central Hospital, Kanazawa 920-8530, Japan; suganumaseigo1978@yahoo.co.jp; 5 Department of Hand Surgery, Skåne University Hospital, 205 02 Malmö, Sweden; 6 Department of Biomedical and Clinical Sciences, Linköping University, 581 83 Linköping, Sweden

**Keywords:** HSP27, diabetes, nerve injury, nerve repair, DRG

## Abstract

We investigated injury-induced heat shock protein 27 (HSP27) expression and its association to axonal outgrowth after injury and different nerve repair models in healthy Wistar and diabetic Goto-Kakizaki rats. By immunohistochemistry, expression of HSP27 in sciatic nerves and DRG and axonal outgrowth (neurofilaments) in sciatic nerves were analyzed after no, immediate, and delayed (7-day delay) nerve repairs (7- or 14-day follow-up). An increased HSP27 expression in nerves and in DRG at the uninjured side was associated with diabetes. HSP27 expression in nerves and in DRG increased substantially after the nerve injuries, being higher at the site where axons and Schwann cells interacted. Regression analysis indicated a positive influence of immediate nerve repair compared to an unrepaired injury, but a shortly delayed nerve repair had no impact on axonal outgrowth. Diabetes was associated with a decreased axonal outgrowth. The increased expression of HSP27 in sciatic nerve and DRG did not influence axonal outgrowth. Injured sciatic nerves should appropriately be repaired in healthy and diabetic rats, but a short delay does not influence axonal outgrowth. HSP27 expression in sciatic nerve or DRG, despite an increase after nerve injury with or without a repair, is not associated with any alteration in axonal outgrowth.

## 1. Introduction

Several factors influence the outcome of nerve repair or reconstruction after a peripheral nerve injury [1]. However, although extensively investigated, the specific mechanisms that regulate death, survival, or regeneration of the injured cells are still not clarified. In particular, regulation of degeneration and regeneration processes is of crucial interest in subjects with diabetes, who often develop neuropathy, and also in subjects after a nerve trauma [2,3,4]. Diabetes continues to increase globally, and the disease especially has effects on peripheral nerves. The potential mechanisms of de- and regeneration in subjects with diabetes have been studied—for instance, after nerve compression lesions [5] and after injury [3]—but the exact mechanisms remain unclear. Finding new clues as to how the regeneration proceeds in subjects with diabetes is therefore still relevant and should be highlighted with elucidating potential protection mechanisms and substances.

Unfortunately, very few substances have been shown to have an effect on regeneration when used as a pharmacological complement to surgery after nerve trauma [6]. From the perspective of surgical repair and reconstruction, the timing of surgery is important for an optimal outcome. A delayed nerve repair, due to delay of consultation, diagnosis, or treatment, may lead to impaired functional recovery compared to if an injured nerve is immediately repaired [7,8]. One reason why an adequate outcome is difficult to achieve after a delayed nerve repair is the inability of Schwann cells (SCs) to support axonal outgrowth based on their reduced ability to produce relevant growth factors [9,10].

A multitude of signaling pathways are associated with a peripheral nerve injury, and these are also of relevance to a delayed nerve repair. Nearly 400 signaling pathways connected to 39 transcription factors have been reported to be involved in control of the regenerative response [11] and axonal outgrowth [12,13,14,15].

Heat shock proteins (HSPs) are classified into five families according to their molecular size: HSP100, HSP90, HSP70, HSP60, and small HSPs (15–30 kDa) including HSP27, the last of which also exists in neurons and Schwann cells [16]. Under physiological conditions, HSP27 is expressed in low amounts in glial cells in the central nervous system, in spinal motor nuclei, and in primary sensory neurons, as well as in central processes of the dorsal root ganglia (DRG) neurons [17]. HSP27 is the only member of the HSP family, which is upregulated in sensory neurons by axotomy [17]. Most HSPs provide strong cytoprotective effects induced by different kinds of stress [18,19] and behave as molecular chaperones [20]. In addition, a decreased HSP27 level in blood is related to impaired nerve function and presence of neuropathy in subjects with diabetes [21,22,23]. While it is not clear how the induction of HSP27 in Schwann cells is regulated, it is obvious that retrograde axonal transport of Jun amino terminal kinase (JNK) signaling components in neurons contributes to the injury-induced c-Jun phosphorylation and activating transcription factor 3 (ATF3) induction [24]. Such events have been shown to result in the upregulation of HSP27 [25] with its subsequent anterograde transport to the injury site [17], in the suppression of apoptosis of the injured neurons, and in the direct contribution to axonal regeneration [17,25,26,27,28], which takes place by affecting cytoskeletal elements [16,28,29]. The relation between c-Jun phosphorylation, ATF3 induction, and HSP27 upregulation is considered to have a major impact on Schwann cells to change their phenotype and become repair cells after injury [30,31,32]. Schwann cells in the distal nerve segment with high HSP27 expression support regenerating axons forming linear staining along with neurofilament activity [16]. Thus, HSP27 may play an important role in peripheral nerve regeneration and in diabetes.

Diabetes, nerve repair, and reconstruction can be studied in animal models. The most common way to induce diabetes in animal in vivo studies is to inject streptozotocin, which results in high blood glucose values through a direct toxic effect on beta-cells. This model is limited by the fact that it is likely that some beta-cells are still left producing insulin after the treatment with streptozotocin [33]. Another more stable animal model for studies of diabetes is represented by Goto-Kakizaki (GK) rats, which have been genetically altered to generate moderately increased blood glucose levels [34]. This model is therefore clinically more relevant to study nerve regeneration in diabetes as compared to the streptozotocin model [3]. The literature in the field about how HSP27 affects nerve regeneration in diabetic GK rats with a modest increased blood glucose level is limited.

**Figure 1:**
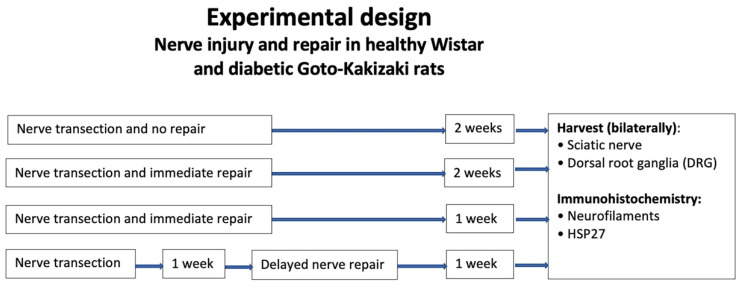
Overview of the experimental design in healthy Wistar and diabetic Goto-Kakizaki rats after unilateral sciatic nerve transection and with or without a nerve repair and two different follow-up time points.

Although immediate nerve repair is partly neuroprotective due to the rapid reconnection of the severed nerve ends, leading to efficient axonal outgrowth in the distal nerve end through the intimate interaction between the axons and Schwann cells [15,35], it is unclear if a delayed nerve repair influences HSP27 dynamics in the distal nerve end or in DRG, and potentially also in diabetes. The purpose of the current study was to investigate how an immediate nerve repair, a delayed nerve repair, and a lack of a nerve repair affect nerve regeneration. In addition, we aimed to investigate if HSP27 expression in the repaired sciatic nerve as well as in DRG was associated with alterations in axonal outgrowth in both healthy and diabetic rats [36]. To study this, we used the two above described healthy and diabetic rat models to measure axonal outgrowth and the expression of HSP27 in sciatic nerves and in DRG after having injured the nerve by transection and left it unrepaired, repaired it immediately after injury, or repaired it after a delay (Figure 1); the unrepaired group is of clinical relevance due to recent reports demonstrating a lack of clinical outcome data supporting any repair of digital nerve injuries in humans [37,38].

Our findings show that an increased HSP27 expression in uninjured sciatic nerves and in their DRGs is associated with diabetic status and that expression increases substantially after nerve injury with and without repair. Injured nerves should appropriately, and also possibly promptly, be repaired irrespective of whether the individual is healthy or diabetic, although diabetes negatively influences axonal outgrowth, but a short delay does not significantly affect outgrowth of axons. However, injury-induced HSP27 expression in the sciatic nerve or in DRG is not associated with any alteration in axonal outgrowth in the present nerve repair models.

## 2. Results

### 2.1. Axonal Outgrowth

**Figure 2:**
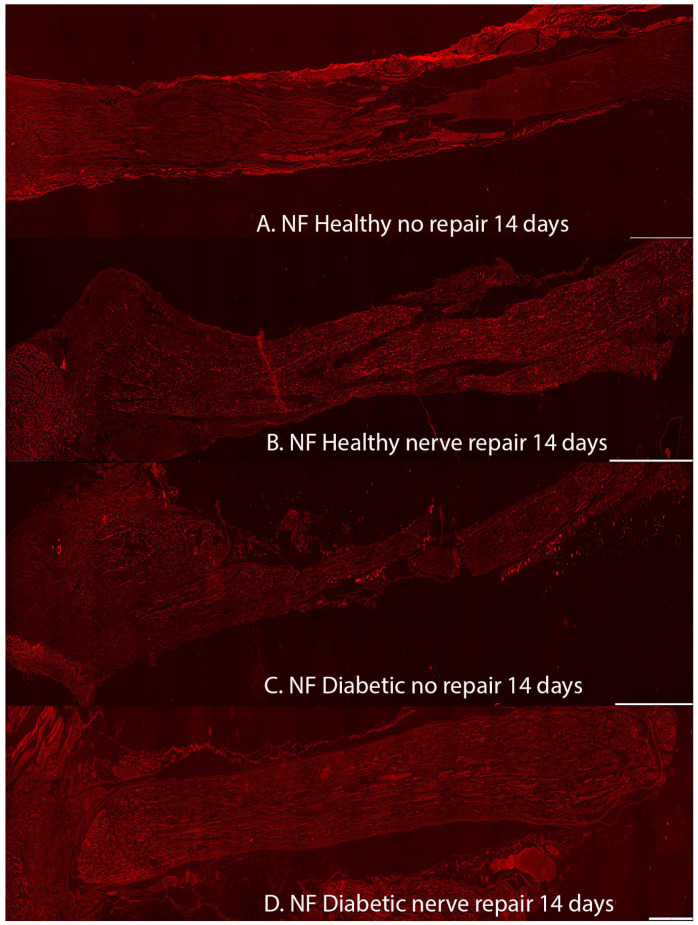
Axonal outgrowth presented as neurofilament staining in the groups in the 14-day injury and repair models from (**A**) healthy Wistar rats with no repair, (**B**) healthy Wistar rats with immediate nerve repair, (**C**) diabetic GK rats with no repair, and (**D**) diabetic rats with immediate nerve repair. All images are taken at 10× magnification. Bar = 1 mm.

**Figure 3:**
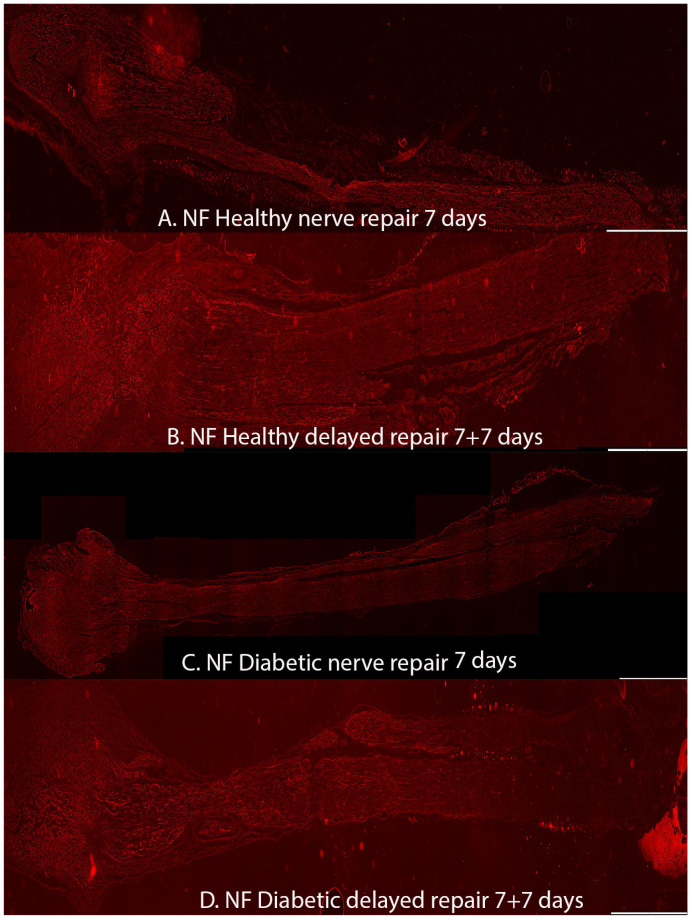
Axonal outgrowth presented as neurofilament staining in the experimental groups in the 7-day follow-up injury and repair models with (**A**) healthy Wistar rats with immediate nerve repair, (**B**) healthy Wistar rats with delayed (7 days) nerve repair, (**C**) diabetic GK rats with immediate nerve repair, and (**D**) diabetic rats with delayed (7 days) nerve repair. All images are taken at 10× magnification. Bar = 1 mm.

**Figure 4:**
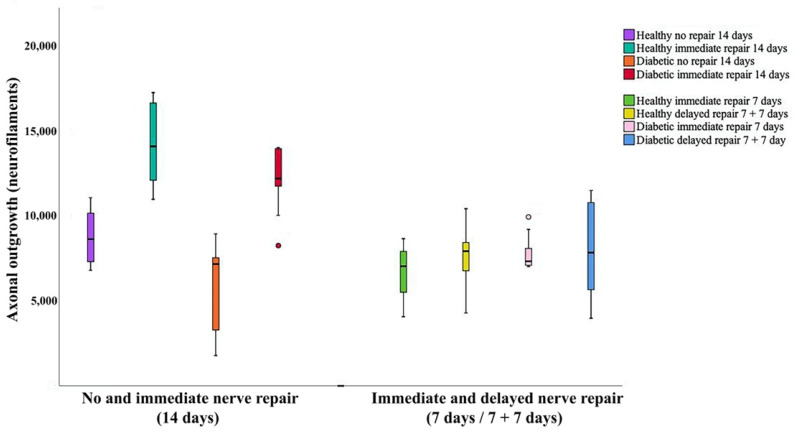
Boxplot of axonal outgrowth (i.e., neurofilament, μm) from the models (i.e., no repair and immediate nerve repair—14-day follow-up, left; immediate and delayed—7 days—nerve repair—7-day follow-up, right) in both healthy Wistar and diabetic GK rats. The box plots represent median values (horizontal line in the middle) and 25th and 75th percentiles (Tukey’s hinge). Error bars show min-max values.

Axonal outgrowth, detected as neurofilament staining, was observed in all groups of rats (Table 1 and Table 2, Figure 2 and Figure 3). As expected, longer axonal outgrowth was observed at 14 days in rats after immediate nerve repair compared to those with the nerve that had been transected without repair (*p* = 0.0001; Kruskal–Wallis test) both in the healthy Wistar and in the diabetic GK rats (*p* < 0.0001; Fisher’s method; Table 1; Figure 4). Outgrowth was, however, superior in the sciatic nerve of healthy Wistar rats compared to the diabetic GK rats (*p* = 0.011; Fisher’s method; Table 1; Figure 4).

No difference in axonal outgrowth was observed between immediate and a delayed nerve repair at a 7-day follow-up, nor was there any difference observed in axonal outgrowth when comparing healthy Wistar and diabetic GK rats from these groups (*p* = 0.43; Kruskal–Wallis test; Table 2; Figure 4).

### 2.2. Expression of HSP27 in Sciatic Nerve

HSP27 expression in the sciatic nerves was measured both at the contralateral uninjured side and at the transected and unrepaired/repaired side, i.e., the latter being done just distal to the injury/repair site (SNL) as well as in the distal nerve segment (SND) at a point where axonal outgrowth had not reached (i.e., from 18–20 mm into the distal nerve end; see Material and Methods).

#### 2.2.1. HSP27 Expression at the Contralateral Uninjured Side of the Sciatic Nerve

**Figure 5:**
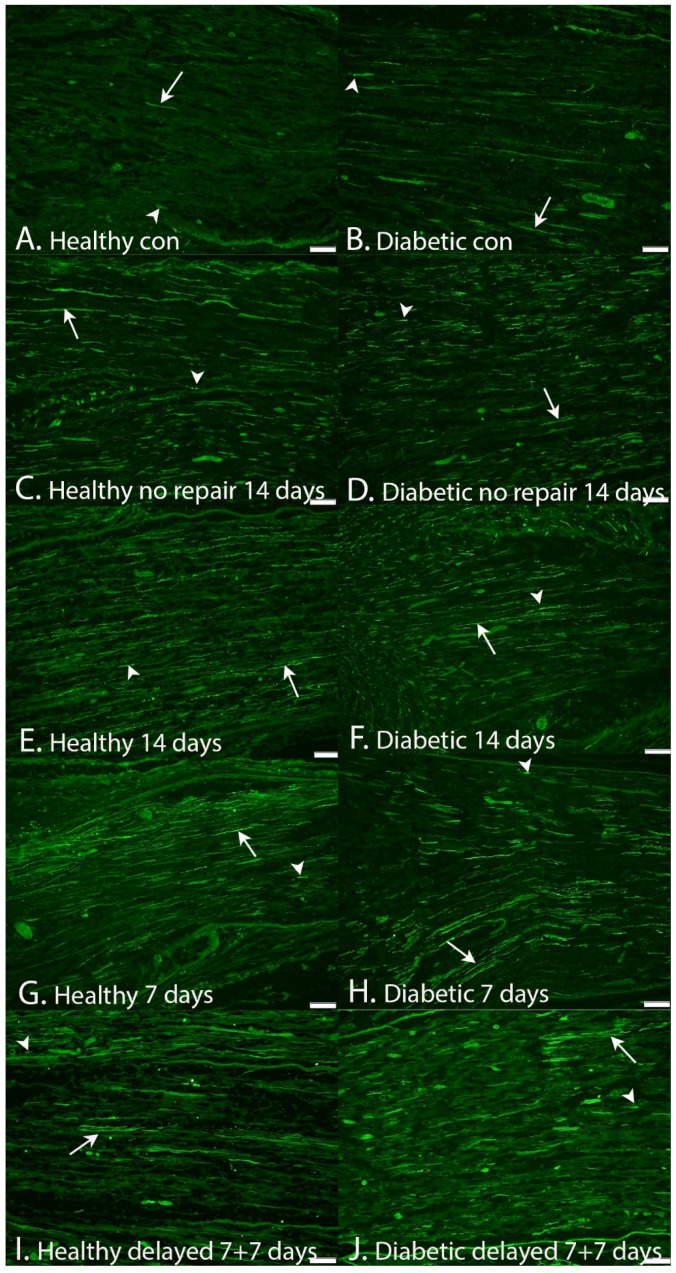
HSP27 expression at the site of lesion (SNL) in the sciatic nerve in healthy (**A**,**C**,**E**,**G**,**I**) and GK diabetic rats (**B**,**D**,**F**,**H**,**J**). (Left panels): (**A**) = contralateral side (14-day model) healthy rats, (**C**) = no nerve repair (14-day follow-up) healthy rats, (**E**) = immediate nerve repair (14-day follow-up) healthy rats, (**G**) = immediate nerve repair (7-day follow-up) healthy rats, and (**I**) = delayed (7 days) nerve repair (7-day follow-up) healthy rats. (Right panels): (**B**) = contralateral side (14-day model) GK rats, (**D**) = no nerve repair (14-day follow-up) GK rats, (**F**) = immediate nerve repair (14-day follow-up) GK rats, (**H**) = immediate nerve repair (7-day follow-up) GK rats, and (**J**) = delayed (7 days) nerve repair (7-day follow-up) GK rats. The arrows mark HSP27 expression in axon(s), and the arrowheads mark the specific Schwann cell (SC) in the images. Bar = 100 μm in all images.

No differences in expression of HSP27 were seen at the contralateral uninjured (control) side of the sciatic nerve in the rats with a 14-day follow-up (*p* = 0.06; Kruskal–Wallis test; Table 1; Figure 5A,B).

In contrast, in the contralateral uninjured (control) side in the models with a 7-day follow-up, a statistical difference of HSP27 expression was found (*p* = 0.008; Kruskal–Wallis test), where the diabetic GK rats had a slightly higher expression than the healthy Wistar rats in the control sciatic nerve (*p* = 0.006; Fisher’s method). However, the type of nerve injury and repair did not affect expression of HSP27 in the sciatic nerve on the control side (*p* = 0.26; Table 2).

#### 2.2.2. HSP27 Expression at the Site of Lesion (SNL) at the Experimental Side

**Figure 6:**
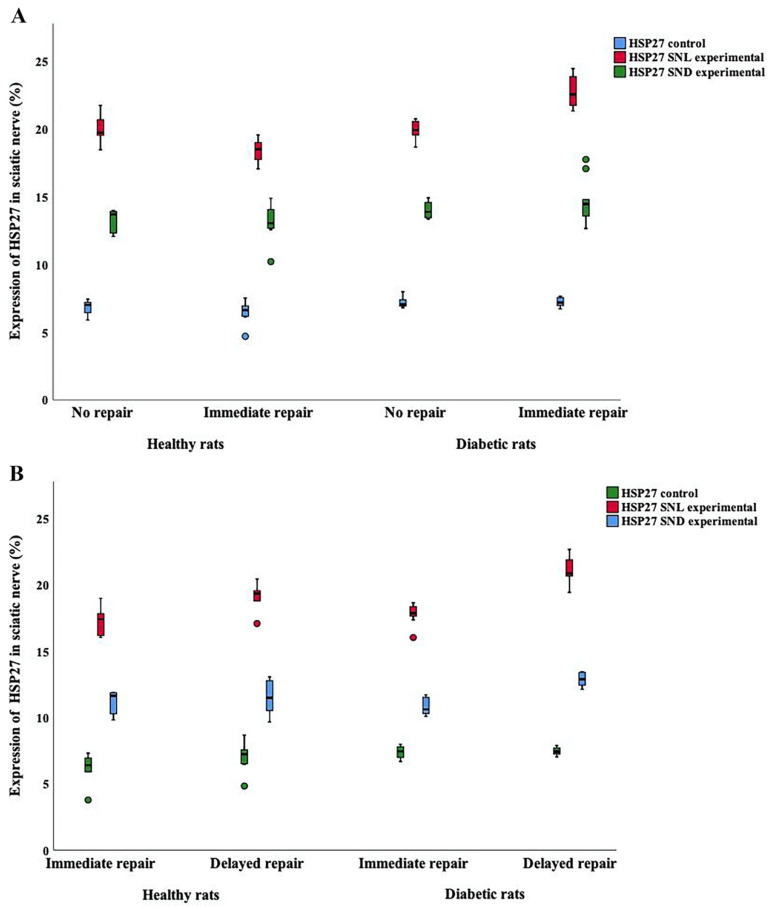
Boxplots demonstrating HSP27 expression in experimental and control sides in the sciatic nerve. (**A**) shows HSP27 expression after no and immediate nerve repair (14-day follow-up) at the site of lesion (SNL), in the distal sciatic nerve (SND) and at the contralateral side both in healthy Wistar and in diabetic GK rats. (**B**) shows the HSP27 expression after immediate and delayed (7 days) nerve repair (7-day follow-up) at the site of lesion (SNL), in the distal part of the nerve (SND) and at the contralateral side both in healthy and in diabetic GK rats. The box plots represent median values (horizontal line in the middle) and 25th and 75th percentiles (Tukey’s hinge). Error bars show min-max values.

Expression of HSP27 was higher in the sciatic nerve at the site of lesion (SNL) after the various nerve injuries and repair models compared to the contralateral uninjured side. Differences in expression of HSP27 were found on the injured/repaired side at the site of lesion (SNL) between the two nerve repair models: no nerve repair and immediate nerve repair, with a 14-day follow-up, both in the healthy Wistar and in the diabetic GK rats (*p* = 0.0001; Kruskal–Wallis test; Table 1; Figure 5C–F and Figure 6A). Generally, HSP27 expression showed a complex pattern at the site of lesion in the sciatic nerve after immediate and no nerve repair, depending on health status (*p* < 0.0001; Fisher’s method). Furthermore, the expression of HSP27 was higher in the diabetic GK rats after such an injury with no and immediate repair than in the healthy rats (*p* = 0.001; Fisher’s test; Table 1; Figure 6A).

The expression of HSP27 was also calculated as a ratio between the experimental and control sides in the groups with 14-day follow-up (exp/con ratio; *p* = 0.006; Kruskal–Wallis test; Table 1) to further visualize any change from the control side. Again, a complex pattern of the ratio at SNL after injury with no or immediate nerve repair was seen depending on health status (*p* = 0.0005; Fisher’s method), but the ratio in the sciatic nerve was not different between heathy and diabetic rats (*p* = 0.07: Fisher’s method; Table 1). Thus, based on ratios, the data indicate that a sciatic nerve transection, increased the expression of HSP27 at SNL after 14 days in the healthy and diabetic GK rats compared to the contralateral uninjured control side.

When analyzing the expression in the sciatic nerve at SNL in the other nerve injury and repair models with immediate and delayed nerve repairs with 7-day follow-up, a statistical difference was found in the two rat models (*p* = 0.0001; Kruskal–Wallis test; Table 2, Figure 5G–J and Figure 6B). A delayed nerve repair induced a higher expression at SNL (*p* < 0.0001; Fisher’s method, Table 2) and a higher expression in the diabetic GK rats (*p* = 0.0002; Fisher’s method).

The expression of HSP27, again calculated as the experimental/control ratio to signify any change from control, in the sciatic nerve at SNL after immediate or a delayed nerve repair also showed a statistical difference (*p* = 0.005; Kruskal–Wallis test; Table 2) with higher expression after a delayed nerve repair (*p* = 0.001; Fisher’s method), but the health status of the rats did not influence expression in the sciatic nerve (*p* = 0.12; Fisher’s method, Table 2). Thus, again based on the ratio, a sciatic nerve transection with an immediate or delayed nerve repair gave an increased expression of HSP27 at SNL after 7 days in both the healthy and diabetic GK rats, irrespective of timing of the repair, compared to the contralateral uninjured control side, with a similar increased expression irrespective of health status.

#### 2.2.3. HSP27 Expression in the Distal Sciatic Nerve (SND) in the Experimental Side

Again, there was an increased expression of HSP27 in the distal sciatic nerves (SND) after the various nerve injuries and repair models compared to the contralateral uninjured sciatic nerve. There were overall significant differences between no nerve repair models and immediate nerve repair models at 14-day follow-up at SND (*p* = 0.048; Kruskal–Wallis test; Table 1; Figure 6A), but with no clear difference concerning nerve injury model (*p* = 0.81; Fisher’s method) and health status (*p* = 0.05; Fisher’s method).

When the expression of HSP27 at SND was calculated as a ratio (exp/control) with a 14-day follow-up to reveal any change after injury, no significant differences at SND were found (*p* = 0.66; Kruskal–Wallis test; Table 1). Thus, in accordance with the data at SNL, the ratio data at SND indicate that a sciatic nerve transection with no or immediate nerve repair increased the expression of HSP27 at SND after 7 days in the healthy and diabetic GK rats compared to the contralateral uninjured control side. However, the ratios at SND were significantly lower than the corresponding data at SNL (*p* < 0.0001; Wilcoxon signed rank test; *n* = 40), indicating a higher expression of HSP27 at the site where outgrowing axons were present in conjunction with the Schwann cells (i.e., SNL) compared to more distally (SND).

When expression of HSP27 was analyzed at SND in the other nerve injury and repair models with immediate and delayed nerve repair and a 7-day follow-up, the repair and health status models differed overall (*p* = 0.0001; Kruskal–Wallis test; Table 2; Figure 6B). The subsequent analysis showed a complex pattern with expression in the healthy and diabetic GK rats (*p* = 0.0003; Fisher’s method) and the type of nerve injury and repair, i.e., immediate and delayed nerve repair with 7-day follow-up (*p* = 0.0009; Fisher’s method; Table 2).

The expression of HSP27 at SND in these nerve injury and repair models was also calculated as the experimental/control ratio to envision any change from the control. Such a calculation showed a significant difference among the groups (*p* = 0.009; Kruskal–Wallis test; Table 2), and with a similar complex pattern of expression in healthy and diabetic GK rats (*p* = 0.04; Fisher’s method) and nerve repair models (*p* = 0.0006; Fisher’s method; Table 2). Hence, a sciatic nerve transection with an immediate or a delayed nerve repair gave a higher expression of HSP27 at SND at the experimental side compared to the contralateral uninjured control side (see ratio; Table 2) after 7 days in the healthy and diabetic GK rats. Again, the ratios at SNL in these nerve repair models, i.e., immediate and delayed nerve repair, were significantly higher than at SND (*p* < 0.0001; Wilcoxon signed rank test; *n* = 40), indicating the impact of the presence of outgrowing axons on the expression of HSP27.

#### 2.2.4. Co-Localization of HSP2 in Schwann Cells and Axons

**Figure 7:**
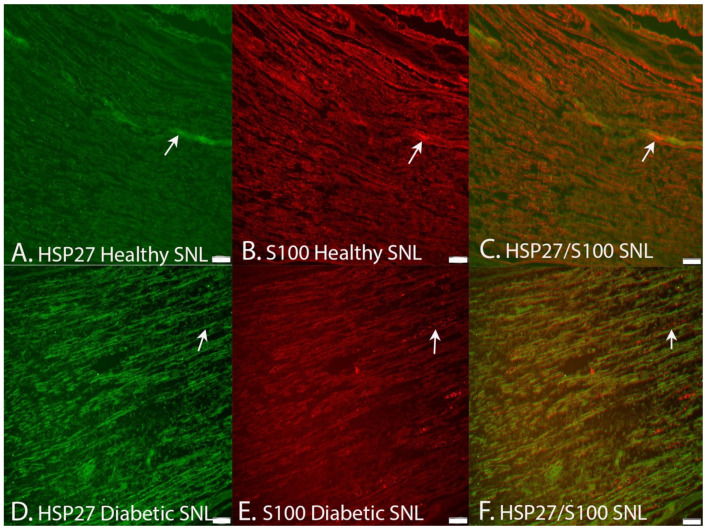
Images demonstrating double staining showing that HSP27 expression (arrows in left panels) is presented in S100-stained Schwann cells (arrows in middle panels) at the site of lesion (SNL) in healthy Wistar and diabetic GK rats. The images (**A**,**B**) are merged together in (**C**). The images (**D**,**E**) are merged together in (**F**). Bar = 100 μm.

**Figure 8:**
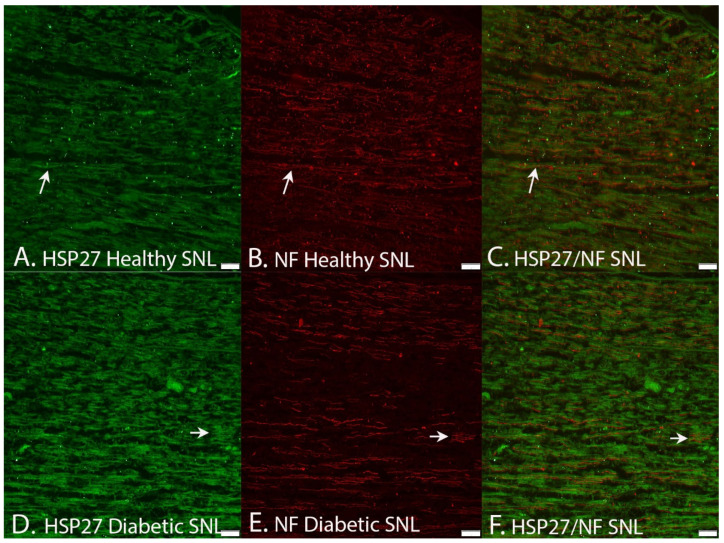
Images demonstrating double staining HSP27 (arrows in left panels) and neurofilaments showing that HSP27 expression is presented in neurofilament-stained axons (middle panels) at the site of lesion (SNL) in healthy Wistar and diabetic GK rats. The images (**A**,**B**) are merged together in (**C**). The images (**D**,**E**) are merged together in (**F**). Bar = 100 μm.

Double staining of HSP27 and S-100 (Figure 7) as well as of HSP27 and neurofilaments (Figure 8) revealed a co-localization of HSP27 and the mentioned markers for Schwann cells and axons, respectively, indicating that HSP27 was expressed both in Schwann cells and in axons in the uninjured and injured sciatic nerves.

### 2.3. Expression of HSP27 in DRG

**Figure 9:**
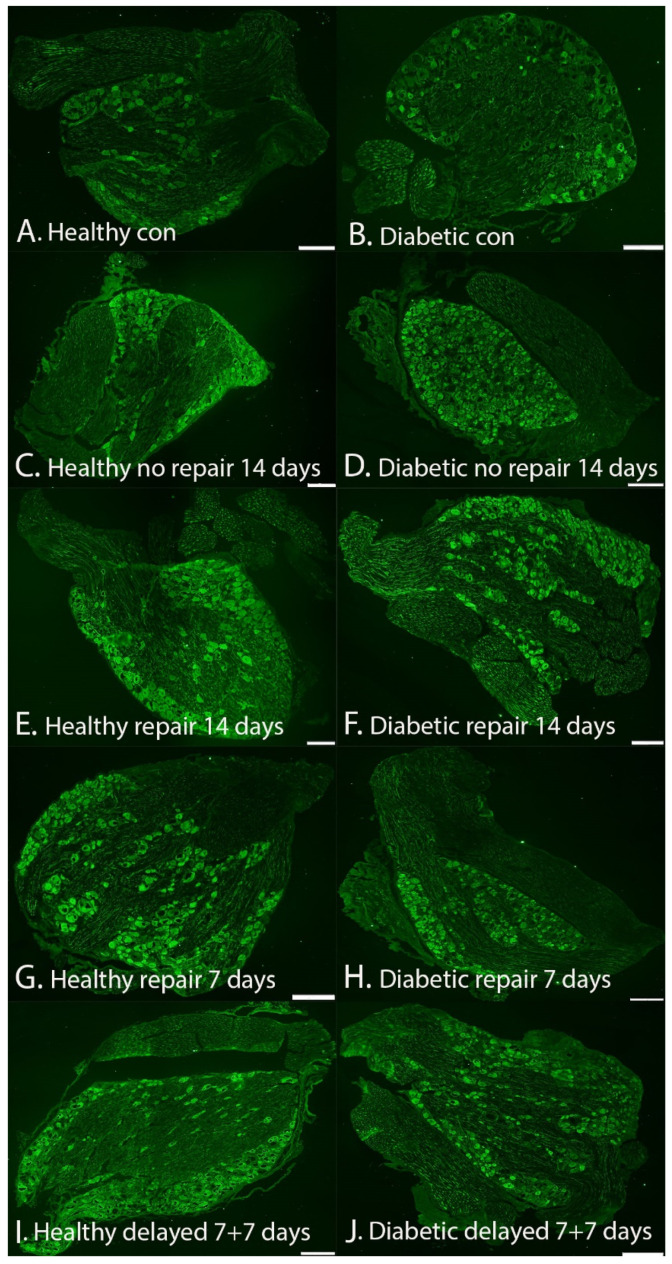
HSP27 expression in the sensory dorsal root ganglion (DRG) in healthy (**A**,**C**,**E**,**G**,**I**) and in GK diabetic rats (**B**,**D**,**F**,**H**,**J**). The left panels: (**A**) = contralateral uninjured side (14-day model) healthy rats, (**C**) = no repair (14-day follow-up) healthy rats, (**E**) = immediate repair (14-day follow-up) healthy rats, (**G**) = immediate repair (7-day follow-up) healthy rats, and (**I**) = delayed (7 days) repair (7-day follow-up) healthy rats. The right panels: (**B**) = contralateral uninjured side (14-day model) GK rats, (**D**) = no repair (14-day follow-up) GK rats, (**F**) = immediate repair (14-day follow-up) GK rats, (**H**) = immediate repair (7-day follow-up) GK rats, and (**J**) = delayed (7 days) repair (7-day follow-up) GK rats. Bar = 200 μm in all images.

HSP27 expression in the DRG was measured both at the contralateral uninjured side (Figure 9A,B) as well as at the transected and unrepaired/repaired side (Figure 9C–J) and was also expressed as a ratio between the experimental and control sides (Table 1 and Table 2).

#### HSP27 Expression in DRG at Contralateral Uninjured and Experimental Sides (i.e., Sensory Neurons)

**Figure 10:**
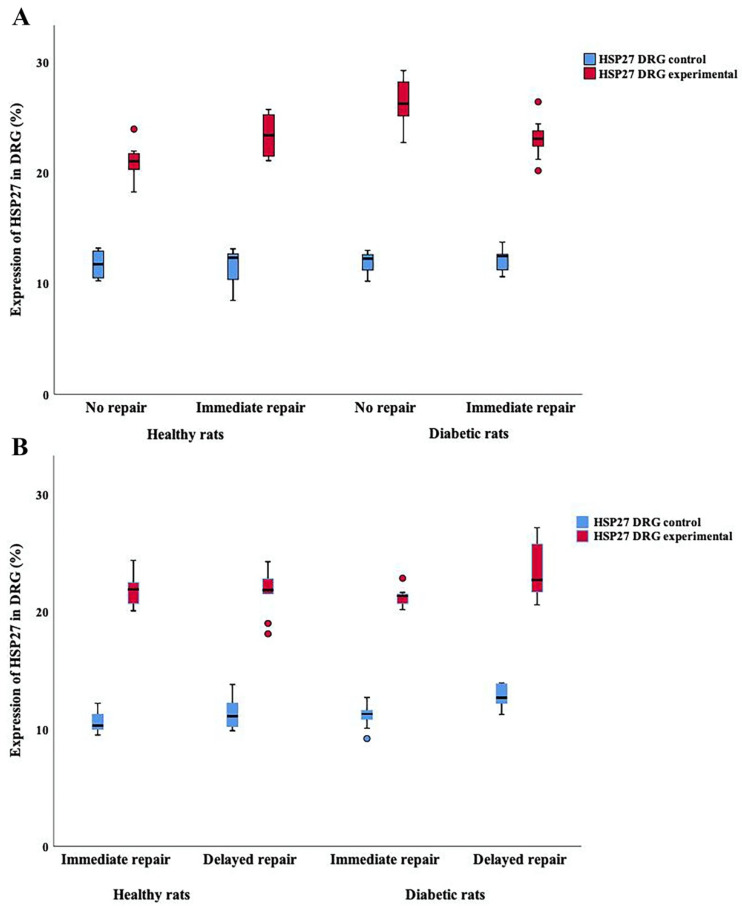
Boxplots demonstrating HSP27 expression from control and experimental sides in the dorsal root ganglion (DRG). (**A**) shows the HSP27 expression after no and immediate nerve repair with 14-day follow-up compared to the contralateral side both in healthy Wistar and in diabetic GK rats. (**B**) shows the HSP27 expression after immediate and delayed (7 days) nerve repair with 7-day follow-up compared to the contralateral side in healthy Wistar and in diabetic GK rats. The box plots represent median values (horizontal line in the middle) and 25th and 75th percentiles (Tukey’s hinge). Error bars show min-max values.

The expression was analyzed in DRG and presented in accordance with the method used for sciatic nerves. In the group with no and immediate nerve repair with 14-day follow-up, no significant differences were observed concerning expression of HSP27 in contralateral control DRG in the healthy Wistar or the diabetic GK rats (*p* = 0.85; Kruskal–Wallis test; Table 1; Figure 9A,B and Figure 10A). However, at the experimental side (*p* = 0.0001; Kruskal–Wallis test), a complex pattern was seen in expression of HSP27 concerning health status (*p* = 0.0008; Fisher’s method) and nerve injury and repair models (*p* = 0004; Fisher’s method; Table 1; Figure 9C–F). The same pattern was observed in a ratio between the experimental and control sides (*p* = 0.008; Kruskal–Wallis test; Table 1; nerve injury model *p* = 0.01 and health status *p* = 0.0004; Fisher’s method). The experimental/control ratio in DRGs, calculated to imply any change from control, indicated that the nerve injury and repairs, irrespective of health status, increased expression of HSP27 in DRG (Figure 10A).

In the other nerve injury and repair models, i.e., immediate and delayed nerve repair with a 7-day follow-up, statistical differences could be found concerning expression of HSP27 in the uninjured control DRG between the healthy Wistar and diabetic GK rats (*p* = 0.001; Kruskal–Wallis test; Figure 10B) with signs of higher expression in rats with a contralateral delayed nerve procedure and with health status (*p* = 0.0002 and 0.012, respectively; Fisher’s test; Table 2), indicating a higher expression if a contralateral delayed procedure was performed and in diabetic GK rats. On the experimental side with immediate and delayed nerve repairs, significant differences were observed (*p* = 0.047; Kruskal–Wallis test; Table 2; Figure 9G–J) with signs of higher expression after delayed nerve repair (nerve injury models *p* = 0.047) but without differences concerning health status (*p* = 0.27; Fisher´s method; Table 2; Figure 9G–J). However, the ratios (experimental/control, i.e., change from control) did not show any differences (*p* = 0.32; Kruskal–Wallis test) with respect to nerve injury model or diabetes, but data indicated that nerve injury and repair increased expression on the experimental side compared to the contralateral uninjured control side (Table 2; Figure 10B).

### 2.4. Regression Analyses and Correlations

#### 2.4.1. Axonal Outgrowth in Association with Type of Nerve Repair, Diabetes Status, and Expression of HSP27

Linear regression analysis was applied to investigate any association between the length of axonal outgrowth after nerve injury with either no or immediate nerve repair with a 14-day follow-up, health status, and expression of HSP27 in nerve or DRG (calculated as ratios). It was found that presence of a diabetic status was associated with an impaired length of axonal outgrowth (unstandardized Beta −2.8 [95% CI −4.3–−1.4]; *p* < 0.0001). Immediate nerve repair, compared to the model where the injury was left untreated (i.e., no nerve repair), was, on the other hand, associated with an increased length of axonal outgrowth (unstandardized Beta 5.5 [95% CI 4.1–7.0]; *p* < 0.0001). The expression of HSP27 (expressed as ratios), neither at any of the examined sites in the sciatic nerve (SNL and SND) nor in the DRG, had any influence on axonal outgrowth (*p* = 0.19, 0.76, and 0.30, respectively).

In the other nerve injury and repair models, i.e., immediate and delayed nerve repair with a 7-day follow-up, no predictive factors for length of axonal outgrowth could be detected by the linear regression analysis as in the other nerve injury models (*p* = 0.29); thus, axonal outgrowth was not affected by health status, nerve repair technique, or expression of HSP27 (ratios) in the sciatic nerve (SNL or SND) or in DRG.

#### 2.4.2. Expression of HSP27 in the Contralateral Uninjured Sciatic Nerve and in DRG Associated to Diabetes Status

A linear regression analysis was also used to analyze if expression of HSP27 in the sciatic nerve or in DRG on the contralateral uninjured side (*n* = 80 in both analyses) was associated with diabetic status. It was found that an increased expression of HSP27 in the contralateral uninjured sciatic nerve was associated with diabetes (unstandardized Beta 0.6 [95% CI 0.3–0.9]; *p* < 0.0001), and an increased expression of HSP27 in the contralateral uninjured DRG also showed an association with diabetes (unstandardized Beta 0.6 [95% CI 0.1–1.2]; *p* = 0.016).

#### 2.4.3. Correlation between Axonal Outgrowth and Expression of HSP27 in the Sciatic Nerve and in DRG

**Figure 11:**
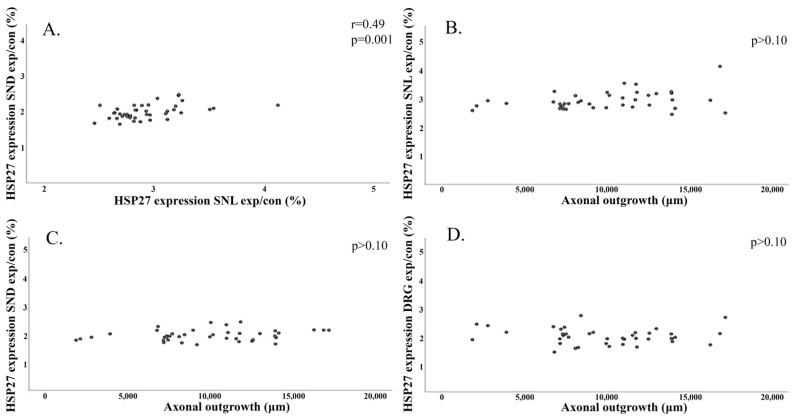
Scatter plots for HSP27 expression (exp/con ratio; %) after no and immediate nerve repair in the models with 14-day follow-up corresponding to Table 1. (**A**) shows a positive correlation for HSP27 expressions (exp/con ratio; %) in the sciatic nerve between the locations SNL and SND. (**B**–**D**) show scatter plots with no correlations between HSP27 expressions (exp/con ratio; %) in the sciatic nerve at SNL (**B**), at SND (**C**), and in DRG (**D**) with length of axonal outgrowth (µm) in the sciatic nerve. *p*- and *r*-values (if significant) are indicated.

Correlations between axonal outgrowth and expression of HSP27 (ratio) were investigated in the two follow-up models (14- and 7-day follow-up; corresponding to Table 1 and Table 2 respectively). However, no correlations between HSP27 expression, expressed as the described ratios (experimental/control), were observed in the nerve (SNL and SND) or in the DRG to the length of axonal outgrowth (*p* > 0.10) after no or immediate nerve repairs with a follow-up of 14 days. Figure 11B–D shows the expression of HSP27 (ratio) with axonal outgrowth in the models with 14-day follow-up; however, HSP27 expression (ratios) at SNL positively correlated with SND (r = 0.49; 0.001; Figure 11A).

**Figure 12:**
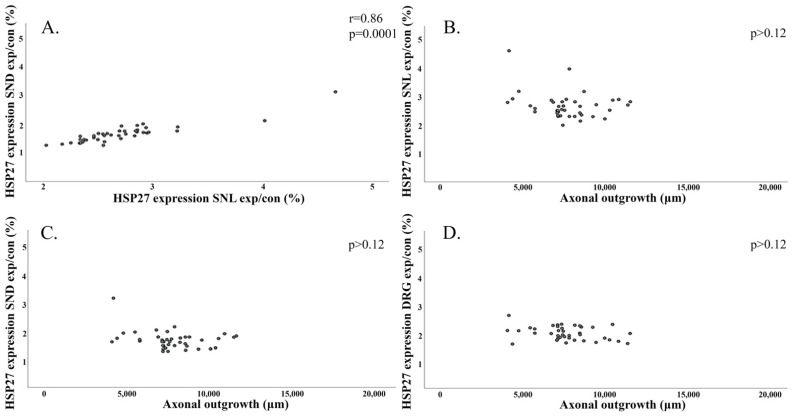
Scatter plots for HSP27 expression (exp/con ratio; %) after immediate and delayed (7 days) nerve repairs in the models with 7-day follow-up corresponding to Table 2. (**A**) shows a positive correlation for HSP27 expression (exp/con ratio; %) in the sciatic nerve at the locations SNL and SND 7 days after the nerve repair. (**B**–**D**) show scatter plots with no correlations between HSP27 expressions (exp/con ratio; %) in the sciatic nerve at SNL (**B**), at SND (**C**), and in DRG (**D**) with length of axonal outgrowth (µm). *p*- and *r*-values (if significant) are indicated.

There were no correlations between expression of HSP27 (ratios) in the nerve (SNL or SND) or in DRG with length of axonal outgrowth after immediate and delayed nerve repair with a 7-day follow-up (*p* > 0.12; Figure 12B–D). The expression of HSP27 (ratio) at SNL again correlated with SND at the 7-day follow-up (r = 0.86; *p* = 0.0001; Figure 12A).

No other correlations were found.

## 3. Discussion

The present study shows that HSP27 is normally expressed in uninjured rat sciatic nerves as well as in corresponding DRG, and an increased HSP27 expression is associated with diabetic status. The HSP27 expression is markedly increased after various nerve injury and repair models in both healthy and diabetic GK rats. More importantly, the increased expression of HSP27 was not, in either the sciatic nerve or DRG, associated with any alteration in axonal outgrowth in the various nerve repair models. Our findings indicated that axonal outgrowth was longer after immediate nerve repair than without a nerve repair both in healthy and in diabetic rats and that diabetes was associated with a decreased axonal outgrowth. We used two adult rat models with four different clinically relevant nerve injury and repair models to measure axonal outgrowth and to evaluate expression of HSP27 in the sciatic nerve and in the DRG. Data from other studies, using other nerve injury and reconstruction models in the sciatic nerve with longer follow-up in healthy and diabetic GK rats, overall indicate a fast as well as prolonged upregulation of HSP27 in DRG [39,40]. The role of HSP27 in neuroprotection has been established, but it has been implicated that HSP27 itself may not be sufficient to affect axonal outgrowth [36]. The finding that an immediate nerve repair after injury gave a longer axonal outgrowth, compared to a model where the nerve was transected and the proximal and distal nerve ends just allowed to slip away from each other in a limited fashion, as in a clinically unrepaired injury, supports the notion that repair of injured digital nerves in humans should be performed [37,38]. A nerve transection lesion should probably not be left unrepaired as recently claimed, a statement which was argued due to a lack of data particularly concerning one specific recovered sensory function, i.e., two-point discrimination, after a digital nerve injury [37,38]. Thus, there is usable support for an immediate nerve repair in both healthy and diabetic subjects, as in the present study, even if a short follow-up was presently used. An unrepaired digital nerve injury may induce a neuroma with risk for substantial symptoms and disability needing further surgery [41].

The control mechanisms of regeneration after nerve injury are complex [42] with a number of molecular players that interact in the neurons as well as in the Schwann cells. One of the players, HSP27, a neuroprotective substance [17,25,26,30], was selected as a marker in the present study to relate to diabetic status as well as to various nerve injury models with the intention that it may be involved in the control of axonal outgrowth after injury in both healthy and diabetic GK rats. The double staining with HSP27 and S-100 as well as with neurofilaments indicated that HSP27 was expressed both in Schwann cells and in axons, respectively. A limitation is that we did not evaluate HSP27 at the RNA level, but our intention was to analyze expression of HSP27 and its effect on axonal outgrowth after a variety of nerve injury and repair models without evaluating gene expression. A study focusing on gene expression would be valuable to perform in the future. An enhanced expression of HSP27 is reported to correlate with axonal regeneration in mature retinal ganglion cells, indicating a growth-associated role of HSP27 in neurons from the central nervous system [43]. Indirect and direct support for promotion of nerve regeneration has also been presented in other nerve injury models and in chemotherapy-induced neuropathy models in vitro and in vivo [17,28,31,44,45,46].

An increased expression of HSP27 was generally found to be slightly associated with diabetes in the uninjured control sciatic nerve as well as in the DRG, which is both in agreement and in contrast to findings from mice with streptozotocin-induced diabetes and Zucker diabetic fatty (ZDF) rats, resembling type 2 diabetes, as well as data in human subjects with and without type 1 and 2 diabetes, where nerve function is also preserved better in those with higher neuronal and blood levels of HSP27 [21,22,23,47]. The data may reflect that the protection mechanism(s) are also present in uninjured sensory nerves and in DRG in diabetic GK rats, where a moderately increased blood glucose is observed, despite a short duration of diabetes. HSP27 is normally anterogradely transported in axons and much weaker retrogradely transported [24] and is present in Schwann cells as well, which is also indicated by the present double staining experiments. The expression of HSP27 was generally higher in the injured sciatic nerve, both at the site of lesion (SNL) as well as in the distal nerve end (SND), in healthy and in diabetic GK rats after the various nerve injury and repair models, indicating compensatory protection mechanism(s) after injury, but such an increase was not reflected in any improved axonal outgrowth as indicated by the regression and correlation analyses. Thus, the mechanism(s) by which HSP27 works may be more related to survival of neurons and not entirely to expression in the proliferating Schwann cells or to axonal outgrowth after injury [17,25]. This is interesting in view of the regulation of HSP27 in neurons, which is believed to be based on activation of JNK and ATF3, factors that together with extracellular signal-regulated kinase (ERK)1/2 and c-jun are associated with proliferation of Schwann cells after injury [24,25,48]. It has been suggested that expression of HSP27 itself is not enough to stimulate axonal outgrowth, which is in accordance with our study [36]. A limitation of the study—although the specific question was not included in our initial aims—and in view of findings that no association was observed between the injury-induced HSP27 expression and any alteration in axonal outgrowth, is that we did not examine if the increase in HSP27 in the sciatic nerve after the nerve injuries is related to a local synthesis or a translocation of existing protein pools: for instance, from the soma into the axon. This has been focused on in other publications from our research group, using a ligation injury in female rats, i.e., a variation of a complete nerve injury, showing anterograde axonal transport of HSP27, which required activation of c-jun and induction of ATF3 in the nucleus [24]. Thus, it is conceivable that the same mechanism is responsible for the present observed increased expression of HSP27 in DRG [17,26], as well as in the sciatic nerve [24]. Accordingly, the higher expression of HSP27 at SNL compared to SND, where axons have not yet reached at the present time points, is most probably explained by the presence of axons at SNL, since Schwann cells are reported to express lower HSP27 levels in an injured nerve [24]. Detailed mechanisms by which HSP27 acts in the distal axon as well as in non-neuronal cells (i.e., Schwann cells) have been described (see, for example, Ma et al. [31], Hirata et al. [16], and Williams et al. [28], respectively).

The expression of HSP27 correlated with the two locations in sciatic nerve, i.e., close to the injury (SNL) and in the distal nerve end (SND). Interestingly, the ratios of expression of HSP27 between the experimental and control sides in the sciatic nerve were higher at SNL than at SND, which may indicate that axonal outgrowth and the presence of neurofilaments close to the Schwann cells did influence the expression of HSP27 locally in the nerve at 7 and 14 days. This may be both related to the anterograde transport of HSP27 as well as to upregulation in the Schwann cells due to a close interaction between these cells and the advancing axons.

We found an increase in HSP27 expression in DRG (i.e., increased ratio between experimental/control sides) after injury in the various nerve injury models, but our observations did not indicate that such an expression of HSP27 in DRG was associated with any alteration in length of axonal outgrowth, neither in the healthy nor in the diabetic GK rats, despite earlier reported anterograde transport of HSP27 from the sensory neurons in DRG [17,24,31]. The findings of an increased ratio of HSP27 in DRG, which most probably is related to survival of sensory neurons after injury, are in accordance with earlier studies, using, for example, other in vivo and in vitro models with neonatal and adult animals, with and without any injury [17,25,28,31,39,40,45,49]. Only in a limited number of studies of rats after various nerve injury and repair/reconstruction models, and in rats with moderately increased blood glucose levels, as in the diabetic GK rats [49,50], has expression of HSP27 in the sciatic nerve and in DRG been previously analyzed [39,40]. Earlier studies have essentially only used models with very high blood glucose levels that were sustained for longer time periods.

The gold standard in nerve repair is still to perform the surgery after injury as soon as possible. In a delayed nerve repair, the distal nerve segment substantially loses its ability to induce and properly support the regeneration over time, which results in ameliorated functional recovery, for both motor and sensory function, due to diminished reinnervation [12,15]. A delayed nerve repair, longer (>30 days) than the presently used delay, also increases the number of apoptotic, cleaved caspase-3-stained Schwann cells and results in a downregulation of ATF3 in such cells in the distal nerve [12,13]. However, with such a short present time of delay in nerve repair (7 days), which may be considered as rather fast in clinical praxis, we could not detect any impact of the length of axonal outgrowth in any of the two rat models. We have no explanation for a lack of difference in our immediate and delayed nerve repair models with the 7-day follow-up, which is in contrast to other published reports using other evaluation methods, indicating a positive effect of predegeneration of the distal nerve segment on axonal outgrowth [51,52]. Furthermore, we could not observe any association between expression of HSP27 and any alteration in axonal outgrowth after immediate or delayed nerve repair with 7-day follow-up of length of axonal outgrowth, irrespective of health status. This was despite an increased expression of HSP27 in the sciatic nerve and in DRG, expressed as ratios, after injury and the immediate and delayed nerve repairs. In the present study, only female rats were used as experimental animals subjected to several nerve repair models after the nerve injury. One may argue that it may be relevant to examine the expression of HSP27 in rats of both sexes since a difference between female and male rats concerning axonal outgrowth is described [3]. However, there are no indications that expression of HSP27 in the peripheral nervous system is related to sex, diabetes, or estrogen levels [21], although differences concerning such factors may be observed in relation to cancer, protection of atherosclerosis, heart diseases, and brain diseases.

We conclude that an increased expression of HSP27 in nerves and in DRG in uninjured control sides is associated with diabetes. Furthermore, expression of HSP27 in sciatic nerve and in DRG increases substantially after various nerve injury and repair models in healthy and diabetic GK rats. However, the injury-induced expression of HSP27 locally in the nerve or in DRG after injury is not associated with any alteration in axonal outgrowth, neither in healthy nor in diabetic GK rats in the various nerve repair models. Injured sciatic nerves should appropriately, and promptly, be repaired irrespective of health status of the subject, although a short delay of 7 days does not influence axonal outgrowth in the short term. Diabetes in a rat model with moderately increased blood glucose levels is negatively associated with axonal outgrowth.

## 4. Materials and Methods

### 4.1. Animals and Surgery

The experiments were approved by the local ethics committee for animal research at Lund University and carried out according to the European Communities Council’s directive regarding care and use of animals for experimental procedures [53]. Two groups of animals were included in the study: one group of healthy female Wistar rats (*n* = 36; Janvier Labs, France) with normal blood glucose levels and one group of diabetic female Goto-Kakizaki (GK) rats (*n* = 44) with a modestly increased blood glucose value (around 7–13 mmol/L). The healthy animals weighed approximately 250 g, and the diabetic GK female rats (breeding at Lund University) had a weight between 180 and 300 g. The age of the rats, both Wistar and GK rats, was approximately around 4–6 months when operated on. The healthy and the diabetic GK animals were divided into four different subgroups: (i) no nerve repair (14-day follow-up), (ii) immediate nerve repair with 7-day follow-up, (iii) immediate nerve repair with 14-day follow-up, and iv) delayed nerve repair (7-day delay before repair and then 7-day follow-up; Figure 1). The animals were kept on a 12 h dark-light cycle with ad libitum feeding.

The rats were anaesthetized with an intraperitoneal injection of a mixture of Rompun^®^ (20 mg/mL; Bayer Health Care, Leverkusen, Germany) and Ketalar^®^ (10 mg/mL, Pfizer, Helsinki, Finland) with a dose of a 2 mL Ketalar^®^ and 0.5 mL Rompun^®^ per 100 g body weight. Postoperatively, all rats were treated with the analgesic Temgesic^®^ in a dose of 0.01–0.05 mg/kg (0.3 mg/mL; Schering-Plough Europe, Brussels, Belgium). Under anesthesia, the right sciatic nerve was exposed unilaterally at mid-thigh level and transected.

In the group without nerve repair (healthy *n* = 10 and diabetic GK rats *n* = 12), the sciatic nerve was transected by scissors unilaterally and the nerve ends were allowed to spontaneously slightly retract as in a non-repaired digital nerve injury [37], and specimens were harvested after 14 days (for harvest of specimens see below). In the group with immediate nerve repair and a 7-day follow-up (healthy *n* = 8 and diabetic GK rats *n* = 12), the nerve was immediately repaired after unilateral transection with three 9-0 Ethilon sutures (Ethicon, Johnson & Johnson, Livingston, UK), and specimens were harvested after 7 days. In the other group with immediate nerve repair and 14-day follow-up (healthy *n* = 8 and diabetic GK rats *n* = 10), the same surgical procedure was performed, and the specimens were harvested after 14 days. In the last group, nerve repair (healthy *n* = 10 and diabetic GK rats *n* = 10) was delayed, i.e., the nerve was unilaterally transected, but left untreated for 7 days and then repaired with sutures, as described above, and harvested after another 7 days. The nerve ends in the last group were repaired with only a slight trimming of the proximal and distal nerve stumps to provide clean nerve ends for the nerve repair. The wounds in all rats were closed after surgery, and the rats were allowed to recover.

### 4.2. Harvest of Specimens

On the designated days for each group, the rats were sacrificed by an intraperitoneal injection of Allfatal vet. Pentobarbital (100 mg/mL) (Omnidea AB, Stockholm, Sweden) followed by heart puncture. Bilaterally, sciatic nerves and DRG (L4 and L5) were dissected in all rats. All the samples were fixed in Stefanini’s fixative (4% paraformaldehyde and 1.9% picric acid in 0.1 M phosphate buffer, pH 7.2) for 24 h. After fixation, the samples were washed in 0.01 M PBS (phosphate-buffered saline, pH 7.4) and kept in 20% sucrose in 0.01 M PBS until processing. For analysis, the samples were embedded in O.C.T. Compound (Histolab products AB, Gothenburg, Sweden) and immediately frozen in a cryostat. All the samples were sectioned longitudinally in 8 μm thickness, mounted on Super Frost^®^ plus slides (Thermo scientific, Braunschweig, Germany), and kept in −20 °C until processing [3,54].

### 4.3. Immunohistochemistry

Sections were air dried and washed in PBS for 15 min and thereafter stained with monoclonal mouse anti-human neurofilament (Dako, Næstved, Denmark) 1:80 in 0.25% Triton-X-100 (Sigma-Aldrich, St Louis, MO, USA) and 0.25% bovine serum albumin (BSA; Sigma-Aldrich, St Louis, MO, USA) in PBS overnight at 4 °C. On the second day, all sections were washed 3 × 5 min with PBS and incubated with the secondary antibody Alexa Fluor 594 goat anti-mouse IgG (Invitrogen, Life Technologies Corporation, Carlsbad, CA, USA) in 1:500 PBS for 2 h at room temperature.

Other sections were air dried and washed in PBS for 15 min and thereafter incubated with HSP27 primary antibodies. Two different antibodies targeting HSP27 were used, as one of them (sc-1048, Santa Cruz Biotechnology, Dallas, TX, USA) was discontinued by the manufacturer during the study. However, no differences in labelling between these antibodies were noticed, according to the intraclass correlation coefficients (ICC), which gave a value between 0.851 and 0.978, considered as good and excellent intra-rater reliability after comparison of these two antibodies [55].

The slides from the diabetic GK rats were incubated with primary goat polyclonal HSP27 antibody (sc-1048, Santa Cruz Biotechnology, Dallas, TX, USA; [24]; Western blotting of antibody presented in Fredricson [56]). The slides from healthy Wistar rats were incubated with rabbit polyclonal HSP27 antibody (ADI-SPA-803, Enzo Life Sciences, Farmingdale, NY, USA). Both antibodies were diluted in 1:200 in 0.25% Triton-X-100 (Sigma-Aldrich, St Louis, MO, USA) and 0.25% bovine serum albumin (BSA; Sigma-Aldrich, St Louis, MO, USA) in PBS overnight at 4 °C.

After overnight incubation with primary antibody, the sections were washed 3 × 5 min with PBS, and the slides from diabetic GK rats were incubated with the secondary Alexa flour 488 donkey anti-goat IgG antibody (Invitrogen, Life Technologies Corporation, Carlsbad, CA, USA) at a dilution of 1:500 in PBS for 2 h at room temperature. The slides from healthy Wistar rats were incubated with the secondary Alexa fluor 488 goat anti-rabbit IgG antibody 1:250 (Invitrogen, Life Technologies Corporation, Carlsbad, CA, USA) at the same time. Finally, after washing, all slides were cover-slipped with Vectashield^®^ mounting medium with DAPI (Vector Laboratories, Burlingame, CA, USA) [57,58].

To visualize HSP27 expression in the Schwann cells and in the axons, double staining with S100 and neurofilament antibodies was performed. Slides from both healthy Wistar and diabetic GK rats were stained with the HSP27 antibody (see above) overnight at 4 °C. The next day, the slides were incubated with the secondary Alexa fluor 488 goat anti-rabbit IgG antibody (1:250; Invitrogen, Life Technologies Corporation, Carlsbad, CA, USA) at room temperature. After 2 h, half of the slides were washed and incubated with mouse monoclonal IgG anti_S100 α/β chain (sc-58839, Santa Cruz Biotechnology Inc., Dallas, TX, USA; diluted 1:300) in 0.25% Triton-X-100 (Sigma-Aldrich, St Louis, MO, USA) and 0.25% bovine serum albumin (BSA; Sigma-Aldrich, St Louis, MO, USA) in PBS overnight at 4 °C. The other slides were incubated with the monoclonal mouse anti-human neurofilament antibody (Dako, Næstved, Denmark; 1:80) in the same blocking solution (see above). On the final day, all slides were washed in PBS and then incubated with Alexa fluor goat anti-mouse IgG (Invitrogen, Life Technologies Corporation, Carlsbad, CA, USA) for 1 h in room temperature, then washed again, and finally the slides were cover-slipped with Vectashield^®^ mounting medium with DAPI (Vector Laboratories, Burlingame, CA, USA).

### 4.4. Photography and Image Analysis

All slides were blindcoded before image analysis started. The length of axonal outgrowth was measured based on neurofilament staining. The nerve specimens from both the control and the experimental sides were analyzed for HSP27 expression. All slides were captured at 10× magnification using a fluorescence microscope, BX63 (Olympus, Tokyo, Japan), equipped with a DP80 digital camera (Olympus, Tokyo, Japan). The digital images were analyzed using the soft image cellSens dimension program (Olympus, Tokyo, Japan).

**Figure 13:**
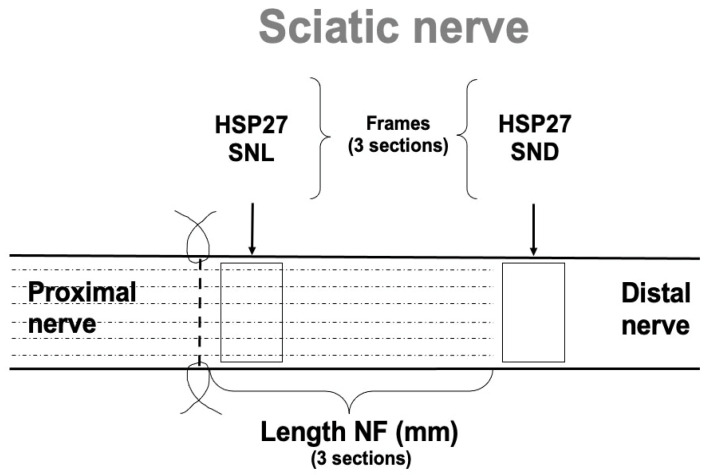
Schematic drawing of the surgically repaired rat sciatic nerve and description of where the specimens were analyzed in healthy Wistar and diabetic GK rats. The location distal nerve segment, i.e., SND, was located distal to the point where the outgrowing axons had reached in any of the rats (i.e., 18–20 mm distal to the injury).

Three randomly selected sections of the nerve from each rat were used for measuring the length of neurofilament protein from the site of lesion and to the front of the longest growing axons, as earlier described [3,12,14]. A mean value from these three sections was calculated as a measure of mean axonal outgrowth. The immunostained area of HSP27 in the contralateral and ipsilateral sides was calculated, both at the site of lesion (SNL) and in the distal part of the nerve (SND) at the injured side, i.e., distal to the point of the longest growing nerve fibers, which was consistently selected at 18–20 mm from the nerve injury (Figure 13).

The HSP27 images were opened in ImageJ 1.52q (a public domain image analysis program developed by Wayne Rasband (retired from NIH), U.S. National Institutes of Health http://imagej.nih.gov/, accessed latest on 26 June 2021) for analysis of the HSP27 expression. In order to estimate the immunofluorescent intensity of the background, the images of the sites SNL and SND in the sciatic nerve were measured in the region of interest (ROI) of 100 × 100 pixels in the endoneurial area furthest away from the transection site. To determine the level of HSP27 expression, the tool called threshold was used, and the intensity threshold was set by adding three times the standard deviation of the background to the mean intensity (+3 × SD). The HSP27-immunostained area of the nerves was then measured across the entire selected section just distal to the site of lesion (SNL) and around 18–20 mm distal to the nerve repair (SND, i.e., distal to the point where the outgrowing axons had reached at the time points in any of the rats) and expressed as percentage of the total area of the section. In the DRG (both L4 and L5), the immunostained area was measured across the whole ganglion, but only in the tissue where sensory neurons were present. The HSP27 expression in the sciatic nerve at the corresponding distance from lesion as well as in DRG was also expressed as a ratio with percent of HSP27 in the experimental side divided by the expression on the control side [3,12,54].

### 4.5. The Intra-Rater Reliability

A calculation of the intraclass coefficient (ICC) by one observer in two different measurements, about one month in between each measurement, gave a value between 0.998 and 0.999, considered to be an excellent reliability [55], meaning that a very high value of agreement of the two measurements of HSP27 expression in ImageJ, made by the same person, but at two different occasions, was achieved.

### 4.6. Statistical Methods

The results are presented as median values (25th–75th percentiles) since the data were considered not to be normally distributed; therefore, non-parametric tests were used for desirable analysis. Kruskal–Wallis (KW) test was used to identify significant differences between the groups and with the Mann–Whitney (MW) test as a post hoc analysis. The results were compared between the no nerve repair group compared to immediate nerve repair group (with 14-day follow-up) as well as the immediate nerve repair group (7-day follow-up) compared to delayed nerve repair group (7-day delay, nerve sutures and then 7-day follow-up). *p*-values were then achieved by using the Fisher´s method for independent samples (Fisher´s combined probability test) based on the chi-squared distribution to identify differences between no nerve repair and immediate nerve repair as well as between immediate nerve repair and delayed nerve repair. In both surgery groups, a comparison was also performed between healthy and diabetic GK rats to detect any difference between surgical methods or health status (Fisher, 1948; https://en.wikipedia.org/wiki/Fisher%27s_method, (accessed latest on 30 April 2021) courtesy of statistician Professor Jonas Bjork, Lund University, Lund, Sweden [3,59]). Wilcoxon signed rank test was used to detect any difference between experimental/control ratios at SNL and SND.

Linear regression analyses were performed to evaluate the influence of predictive factors (i.e., health status, expression of HSP27 (ratio) in sciatic nerve and in DRG) on length of axonal outgrowth (i.e., neurofilament; dependent factor) in the main two nerve injury and repair models (i.e., no and immediate nerve repair with a follow-up of 14 days as well as immediate and delayed (7 days) nerve repair with 7-day follow-up; unstandardized Beta [95% CI]; *p*-value). Correlation was tested between axonal outgrowth and HSP27 expression (ratio) at the two locations (sciatic nerve—SNL and SND—and DRG) as well as of HSP27 expression (ratio) between SNL and SND by using the Spearman correlation test, where an *r*-value > 0.30 (i.e., >0.30–0.7 = moderate correlation; >0.70 = strong correlation) was set as relevant. Statistical analyses were performed using IBM SPSS (version 26). A *p*-value of < 0.05 was considered significant.

## 5. Conclusions

An increased HSP27 expression in nerves and in DRG at the uninjured control side is associated with diabetes. Expression of HSP27 in the injured sciatic nerve and in corresponding DRG substantially increases after injury, but does not seem to be associated with any alteration in axonal outgrowth in the evaluated nerve repair models using adult healthy Wistar and diabetic GK rats. Diabetes is negatively associated with axonal outgrowth. Injury to the sciatic nerve should appropriately and most probably promptly be repaired in healthy and diabetic subjects, but a short delay does not influence axonal outgrowth.

## Figures and Tables

**Table 1:** Axonal outgrowth and expression of HSP27 in uninjured contralateral (control) and injured (experimental), i.e., no or immediate repair (follow-up 14 days), sciatic nerve and corresponding L4 and L5 (pooled data) dorsal root ganglia (DRG) of healthy Wistar and diabetic Goto-Kakizaki rats.

	Healthy Wistar Rats		Diabetic Goto-Kakizaki Rats				
Treatment	No Repair (14 Days) (*n* = 10)	Immediate Repair (14 Days) (*n* = 8)	No Repair (14 Days) (*n* = 12)	Immediate Repair (14 Days) (*n* = 10)	*p*-Values (KW ^a^)	Fisher’s Method ^b^	
						No Repair/Immediate Repair	Healthy Rats/ Diabetic Rats
Axonal outgrowth (mm)	8.6 (7.3–10.4)	14.1 (11.8–16.8)	7.2 (3.0–7.6)	12.2 (11.3–13.9)	**0.0001**	**<0.0001**	**0.011**
HSP27 Sciatic nerve Contralateral control (%)	7.0 (6.3–7.2)	6.6 (6.1–7.0)	7.0 (6.9–7.4)	7.2 (6.9–7.6)	0.06	NA	NA
HSP27 SNL Experimental (%)	19.7 (19.5–20.7)	18.5 (17.4–19.2)	19.9 (19.4–20.6)	22.6 (21.7–23.9)	**0.0001**	**<0.0001**	**0.001**
HSP27 SNL Exp/control ratio	2.8 (2.7–3.2)	2.7 (2.5–3.0)	2.8 (2.7–2.9)	3.2 (3.0–3.2)	**0.006**	**0.0005**	0.07
HSP27 SND Experimental (%)	13.7 (12.3–13.9)	13.0 (12.6–14.3)	13.9 (13.4–14.6)	14.5 (13.4–15.4)	**0.048**	0.81	0.05
HSP27 SND Exp/control ratio	1.9 (1.8–2.0)	2.1 (1.8–2.2)	1.9 (1.8–2.0)	2.0 (1.8–2.2)	0.66	NA	NA
HSP27 DRG Contralateral control (%)	11.7 (10.4–12.9)	12.3 (10.4–12.8)	12.3 (11.2–12.6)	12.5 (11.1–12.8)	0.85	NA	NA
HSP27 DRG Experimental (%)	21.1 (20.0–21.8)	23.4 (21.3–25.3)	26.3 (25.0–28.3)	23.1 (22.1–23.9)	**0.0001**	**0.0004**	**0.0008**
HSP27 DRG Exp/Control ratio	1.7 (1.6–2.1)	2.0 (1.9–2.1)	2.2 (2.0–2.4)	1.9 (1.8–2.1)	**0.008**	**0.01**	**0.004**

Values are median (interquartile range; IQR). DRG = dorsal root ganglion. SNL = sciatic nerve close to the site of lesion. SND = the distal sciatic nerve. ^a^ Kruskal–Wallis with post hoc Mann–Whitney *U*-test for individual groups = statistical difference between appropriate groups used for ^b^ Fisher’s method for independent samples based on the chi-squared distribution (see methods). NA = not applicable.

**Table 2:** Axonal outgrowth and expression of HSP27 in uninjured (control) injured and repaired (experimental), i.e., immediately done or with a delay (7 days) and with a follow-up of 7 days, sciatic nerve and corresponding L4 and L5 (pooled data) dorsal root ganglia (DRG) of healthy Wistar and diabetic Goto-Kakizaki rats.

	Healthy Wistar Rats		Diabetic Goto-Kakizaki Rats				
Treatment	Immediate Repair (7 Days) (*n* = 8)	Delayed Repair (7 + 7 Days) (*n* = 10)	Immediate Repair (7 Days) (*n* = 12)	Delayed Repair (7 + 7 Days) (*n* = 10)	*p*-Values (KW ^a^)	Fisher’s Method ^b^	
						Immediate Repair/Delayed Repair	Healthy Rats/ Diabetic Rats
Axonal outgrowth (mm)	7.0 (5.4–8.2)	7.9 (6.7–8.9)	7.3 (7.1–8.2)	7.8 (5.4–10.9)	0.43	NA	NA
HSP27 Sciatic nerve Contralateral control (%)	6.4 (5.9–7.0)	7.2 (6.5–7.6)	7.5 (7.0–7.8)	7.4 (7.2–7.7)	**0.008**	0.26	**0.006**
HSP27 SNL Experimental (%)	17.4 (16.2–17.9)	19.3 (18.8–19.6)	17.9 (17.6–18.4)	20.9 (20.4–21.9)	**0.0001**	**<0.0001**	**0.0002**
HSP27 SNL Exp/control ratio	2.6 (2.4–3.1)	2.8 (2.5–2.9)	2.4 (2.3–2.6)	2.8 (2.7–2.9)	**0.005**	**0.001**	0.12
HSP27 SND Experimental (%)	11.6 (10.2–11.9)	11.5 (10.5–12.8)	10.6 (10.3–11.5)	12.9 (12.4–13.4)	**0.0001**	**0.0009**	**0.0003**
HSP27 SND Exp/control ratio	1.7 (1.5–2.0)	1.7 (1.4–1.8)	1.5 (1.3–1.6)	1.7 (1.6–1.8)	**0.009**	**0.0006**	**0.04**
HSP27 DRG Contralateral control (%)	10.3 (10.0–11.6)	11.1 (10.2–12.3)	11.3 (10.8–11.6)	12.7 (12.2–13.9)	**0.001**	**0.0002**	**0.012**
HSP27 DRG Experimental (%)	21.9 (20.6–22.5)	21.9 (20.9–23.0)	21.4 (20.7–21.5)	22.7 (21.5–26.0)	**0.047**	**0.047**	0.27
HSP27 DRG Exp/Control ratio	2.1 (1.8–2.2)	1.9 (1.7–2.2)	1.9 (1.8–2.0)	1.9 (1.6–2.0)	0.32	NA	NA

Values are median (interquartile range; IQR). DRG = dorsal root ganglion. SNL = sciatic nerve close to the site of lesion. SND = the distal sciatic nerve. ^a^ Kruskal–Wallis with post hoc Mann–Whitney *U*-test for individual groups = statistical difference between appropriate groups used for ^b^ Fisher’s method for independent samples based on the chi-squared distribution (see methods). NA = not applicable.

## Data Availability

The datasets generated and analyzed during the current study are not publicly available, but data can be available for researchers after a special review that includes approval of the research project.
